# Melatonin modulates the fetal cardiovascular defense response to acute hypoxia

**DOI:** 10.1111/jpi.12242

**Published:** 2015-05-13

**Authors:** Avnesh S Thakor, Beth J Allison, Youguo Niu, Kimberley J Botting, Maria Serón-Ferré, Emilio A Herrera, Dino A Giussani

**Affiliations:** 1 Department of Physiology, Development & Neuroscience, University of CambridgeCambridge, UK; 2 Department of Radiology, Stanford UniversityStanford, CA, USA; 3 Facultad de Medicina, Programa de Fisiopatología, Instituto de Ciencias Biomédicas, Universidad de ChileSantiago, Chile

**Keywords:** cardiovascular, hypoxia, melatonin, nitric oxide, oxidative stress

## Abstract

Experimental studies in animal models supporting protective effects on the fetus of melatonin in adverse pregnancy have prompted clinical trials in human pregnancy complicated by fetal growth restriction. However, the effects of melatonin on the fetal defense to acute hypoxia, such as that which may occur during labor, remain unknown. This translational study tested the hypothesis, in vivo, that melatonin modulates the fetal cardiometabolic defense responses to acute hypoxia in chronically instrumented late gestation fetal sheep via alterations in fetal nitric oxide (NO) bioavailability. Under anesthesia, 6 fetal sheep at 0.85 gestation were instrumented with vascular catheters and a Transonic flow probe around a femoral artery. Five days later, fetuses were exposed to acute hypoxia with or without melatonin treatment. Fetal blood was taken to determine blood gas and metabolic status and plasma catecholamine concentrations. Hypoxia during melatonin treatment was repeated during in vivo NO blockade with the NO clamp. This technique permits blockade of de novo synthesis of NO while compensating for the tonic production of the gas, thereby maintaining basal cardiovascular function. Melatonin suppressed the redistribution of blood flow away from peripheral circulations and the glycemic and plasma catecholamine responses to acute hypoxia. These are important components of the fetal brain sparing response to acute hypoxia. The effects of melatonin involved NO-dependent mechanisms as the responses were reverted by fetal treatment with the NO clamp. Melatonin modulates the in vivo fetal cardiometabolic responses to acute hypoxia by increasing NO bioavailability.

## Introduction

Melatonin is mostly associated with circadian and seasonal rhythmicity, but it is also important in reproduction [1]. Maternal plasma melatonin levels are elevated during pregnancy, reaching a maximum at term and then returning to basal levels soon after delivery [2]. The expression of melatonin receptors in the placenta and fetal tissues further suggests a functional role in feto-placental development [3]. Melatonin is also a powerful antioxidant [4], and the potency of its antioxidant actions relates directly to its combined ability to scavenge free radicals [5], to enhance the expression and/or activity of antioxidant enzymes [6], and to reduce the leakage of electrons from the mitochondrial electron transport chain [7, 8]. We and others have shown that the antioxidant actions of melatonin increase umbilical blood flow and it is protective on the placenta and on fetal cardiovascular function in pregnancy complicated by chronic adverse intrauterine conditions [9–11]. In addition, melatonin has been shown to protect the villous trophoblast against hypoxia-reperfusion-induced oxidative stress and apoptosis [12]. These findings have led to a phase I clinical trial to test the therapeutic efficacy of maternal oral melatonin administration in women with a pregnancy complicated by fetal growth restriction [13]. However, the effects of melatonin on the capacity of the fetus to respond to episodes of acute hypoxic stress, such as that which occurs during labor and delivery, remain completely unknown.

The fetal defense to acute episodes of hypoxic stress is contingent on integrated cardiovascular and metabolic responses to facilitate fetal survival during the period of reduced oxygen availability. The fetal metabolic responses involve an increase in glucose and lactate in the fetal circulation [14]. The cardiovascular defense to acute hypoxia includes bradycardia and peripheral vasoconstriction [15, 16]. The latter aids the redistribution of the fetal cardiac output, and thereby of oxygen and glucose delivery, away from peripheral and toward essential vascular beds, such as the cerebral circulation; this response is also known as the “fetal brain sparing effect” [16]. The physiology of the fetal cardiovascular responses to acute hypoxia is well established. Fetal bradycardia and the peripheral vasoconstriction are triggered exclusively by a carotid chemoreflex [17]. The peripheral vasoconstriction is then maintained by the release of constrictor agents into the fetal circulation, including catecholamines [18, 19]. Recently, we have also made the discovery that a vascular oxidant tone, determined by the balance between the superoxide anion and nitric oxide (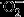: NO), is operational in the late gestation fetus [20]. This oxidant tone acts locally at the level of the fetal vasculature, whereby an increase in the ratio promotes constriction and a decrease favors vasodilatation [9, 21, 22]. Consequently, fetal treatment with agents that increase NO bioavailability opposes chemoreflex and humoral constrictor influences and thereby blunts the fetal peripheral vasoconstrictor response to acute hypoxia [20, 23].

This study tested the hypothesis that melatonin modulates the fetal defense response to acute episodes of hypoxia by affecting NO bioavailability. The hypothesis was tested by investigating the in vivo actions of exogenous melatonin on fetal cardiovascular and metabolic function during acute hypoxic stress using the late gestation ovine fetus as an experimental model. Different doses of melatonin were used to address any dose-dependent actions. Acute hypoxia was induced with and without in vivo NO blockade using an established NO clamp [24–26], to determine which effects of melatonin involved NO-dependent mechanisms. Plasma concentrations of epinephrine and norepinephrine were also measured to establish whether any effects of melatonin on cardiometabolic function involved alterations in circulating catecholamine levels. Melatonin was infused directly into the fetal circulation to isolate its direct effects on the fetus independent of confounding effects on the maternal and/or the placental physiology.

## Methods

### Surgical preparation

All procedures were approved by the Ethical Review Committee of the University of Cambridge. All procedures were performed under the UK Animals (Scientific Procedures) Act 1986 and conform to the Guide for the Care and Use of Laboratory Animals published by the US National Institutes of Health (NIH Publication No. 85-23, revised 1996).

Six Welsh Mountain sheep fetuses were surgically instrumented for long-term recording at 124 ± 1 days of gestation (term is *ca*. 145 days) as previously described in detail [27]. Under general anesthesia (1.5–2.0% halothane in 50:50 O_2_:N_2_O), midline abdominal and uterine incisions were made, the fetal hind limbs were exteriorized, and, on one side, femoral arterial (i.d., 0.86 mm; o.d., 1.52 mm; Critchley Electrical Products, Kingsgrove, NSW, Australia) and venous (i.d., 0.56 mm; o.d., 0.96 mm) catheters were inserted. Another catheter was anchored onto the fetal hind limb for recording of the reference amniotic pressure. A Transonic flow transducer was then positioned around the contra-lateral femoral artery (2R or 3S). The uterine incisions were closed in layers, the dead space of the catheters was filled with heparinized saline (80 i.u. heparin/mL in 0.9% NaCl), and the catheter ends were plugged with sterile brass pins.

### Postoperative care

During recovery, ewes had free access to hay and water and were fed concentrates twice daily (100 g sheep nuts no. 6; H & C Beart Ltd., Kings Lynn, UK). Antibiotics were administered daily to the ewe (0.20–0.25 mg/kg i.m. Depocillin; Mycofarm, Cambridge, UK) and fetus i.v. and into the amniotic cavity (150 mg/kg Penbritin; SmithKline Beecham Animal Health, Welwyn Garden City, Hertfordshire, UK). Following 72 hr of postoperative recovery, ewes were transferred to metabolic crates where they were housed for the remainder of the protocol. The fetal arterial and amniotic catheters were connected to sterile pressure transducers (COBE; Argon Division, Maxim Medical, Athens, TX, USA), and calibrated mean fetal arterial blood pressure (corrected for amniotic pressure) and fetal heart rate (triggered via a tachometer from the pulsitility in the arterial blood pressure signal) were recorded continually using a computerized Data Acquisition System (DAS; Department of Physiology, Cambridge University, Cambridge, UK).

### Experimental protocol

**Fig. 1:**
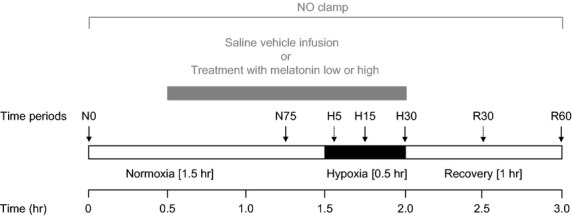
Diagrammatic representation of the experimental protocol. The experimental protocol consisted of a 3-hr period divided into the following: 1.5-hr normoxia, 0.5-hr hypoxia (black bar), and 1-hr recovery, during saline vehicle infusion (n = 6), treatment with melatonin low (0.05 ± 0.01 *μ*g/kg/min; n = 6), treatment with melatonin high (0.5 ± 0.1 *μ*g/kg/min; n = 6), or treatment with melatonin high during nitric oxide (NO) blockade with the NO clamp (n = 6; gray bar). Arrows represent times at which arterial blood samples were collected.

Following at least 5 days of postoperative recovery, all fetuses were subjected to acute hypoxia experiments, carried out on consecutive days in a randomized order (Fig. 1). Each protocol consisted of a 3-hr period divided into 1.5-hr normoxia, 0.5-hr hypoxia, and 1-hr recovery, during a slow i.v. infusion of either heparinized saline vehicle (0.5% ethanol-99.5% heparinized saline), treatment with a low dose of melatonin (0.05 ± 0.01 *μ*g/kg/min), treatment with a high dose of melatonin (0.5 ± 0.1 *μ*g/kg/min), or treatment with melatonin high during NO blockade with the NO clamp (Fig. 1). The doses of melatonin were calculated retrospectively from the fetal weights (3.4 ± 0.3 kg) obtained at *postmortem* and were administered to the fetus having been dissolved in 0.5% ethanol-99.5% heparinized saline (80 i.u. heparin/mL in 0.9% NaCl) solution. The doses were chosen to achieve the lowest plasma concentrations compatible with melatonin having the capacity to act as antioxidant [28, 29]. For comparison, the high dose of melatonin used is *ca*. 32 times lower than that recommended for the prevention of jet lag in humans [30]. The NO clamp is an established technique previously validated in our laboratory that is able to block the production of NO in vivo without affecting basal cardiovascular function [24–26]. In brief, a bolus dose (100 mg/kg dissolved in 2 mL heparinized saline) of L-NAME (N^G^-nitro-l-arginine methyl ester; Sigma Chemicals, Dorset, UK) was injected via the femoral artery, immediately followed by fetal i.v. infusion with sodium nitroprusside (SNP; Sigma Chemicals; 5.1 ± 2.0 *μ*g/kg/min: mean ± 1 S.D.; dissolved in heparinized saline). The infusion rate of SNP was titrated to avoid any perturbation in basal arterial blood pressure. While fetal treatment with L-NAME alone leads to pronounced systemic vasoconstriction and hypertension, combined treatment of the fetus with both L-NAME and SNP compensates for the tonic production of the gas, maintains basal cardiovascular function, and blocks de novo synthesis of NO during stimulated conditions, such as during acute hypoxia. At the end of the experimental protocol, the effectiveness of NO blockade by the NO clamp and the persistence of L-NAME in the system were tested by withdrawal of the SNP infusion. This unmasked the influence of fetal treatment with L-NAME alone and led to a significant increase in arterial blood pressure, a fall in heart rate, and an increase in femoral vascular resistance (FVR) (data not shown).

Acute hypoxic stress in the fetus was induced by maternal inhalational hypoxia for 30 min by changing the concentrations of gases breathed by the ewe to 6% O_2_ in N_2_ with small amounts of CO_2_ (15 L/min air: 35 L/min N_2_: 1.5–2.5 L/min CO_2_) [25]. This mixture was designed to reduce fetal P_*a*_O_2_ to *ca*. 10 mmHg while maintaining fetal P_a_CO_2_. Following the 0.5-hr period of hypoxia, the ewe was returned to breathing air for the 1-hr recovery period. At the end of the experimental protocol, the ewes and fetuses underwent euthanasia with a lethal dose of sodium pentobarbitone (200 mg/kg i.v. Pentoject; Animal Ltd, York, UK). The positions of the implanted catheters and flow probe were confirmed, and the fetuses were weighed.

### Blood sampling and hormone measurements

During the experimental protocol, descending aortic blood samples were taken using sterile techniques from the fetus at set time intervals to determine arterial blood gases and pH (0.3 mL; Fig. 1). Arterial blood gas and pH measurements were made using an ABL5 blood gas analyzer (Radiometer; Copenhagen, Denmark; measurements corrected to 39.5°C for the fetus). Values for percentage saturation of hemoglobin with oxygen (Sat Hb) were determined using a hemoximeter (OSM3; Radiometer). In addition, blood glucose and lactate concentrations were measured by an automated analyser (Yellow Springs 2300 Stat Plus Glucose/Lactate Analyser; YSI Ltd., Farnborough, UK). An additional 2 mL of arterial blood was collected under sterile conditions for hormone analyses at set intervals (Fig. 1, arrows). Samples were centrifuged and the plasma obtained was then dispensed into prelabelled tubes of either chilled heparin tubes (2 mL Li^+^/heparin tubes; L. I. P. Ltd., Shipley, West Yorkshire, UK) containing reduced glutathione (4 nmol per tube; G-4251; Sigma Chemicals) and EGTA (5 nmol per tube; E-4378; Sigma Chemicals) for catecholamine analysis or chilled EDTA tubes (2 mL K^+^/EDTA; L.I.P., Ltd.) for the determination of circulating plasma concentrations of melatonin. All samples were stored at −80°C until analysis. All hormone measurements were performed within 2 months of sample collection.

Fetal plasma concentrations of melatonin were measured using RIA, as previously validated for use in ovine plasma [9]. All samples were analyzed in duplicate at the same time. Plasma samples were extracted with diethyl ether prior to assaying. The assay used melatonin antiserum (batch no. 704 8483; Guildhay Antisera Ltd., Guildford, Surrey, UK) and O-methyl-3H-labeled melatonin (85 Ci mmol/L; Amersham Biosciences AB, SE-751 84, Uppsala, Sweden) tracers. The sensitivity of the assay was 21.5 pmol/L, and the inter- and intra-assay coefficients of variation were both <15%. The melatonin antiserum showed <1% cross-reactivity with N-acetyltryptamine, 6-hydroxy MT, N-acetyltryptophan, and other related amines.

Fetal plasma epinephrine and norepinephrine concentrations were measured by high-pressure liquid chromatography (HPLC) using electrochemical detection as previously described in detail [19, 31]. The samples were prepared by the absorption of 250 *μ*L of plasma onto acid-washed alumina, and 20 *μ*L aliquots of the 100 *μ*L perchloric acid elutes was injected onto the column. Dihydroxybenzylamine was added as the internal standard to each plasma sample before absorption. The limit of sensitivity for the assay was 20 pg/mL for epinephrine and norepinephrine. Recovery ranged from 63% to 97%, and all catecholamine values were corrected for their respective recovery. The interassay coefficients of variation for epinephrine and norepinephrine were 7.3% and 6.2%, respectively.

### Data and statistical analyses

All variables are expressed as mean ± S.E.M. Values for arterial blood gases and pH are expressed at 0 (N0) and 75 (N75) min of normoxia, at 5 (H5), 15 (H15), and 30 (H30) min of hypoxia, and at 30 (R30) and 60 (R60) min of recovery. Values for plasma concentrations of melatonin are expressed at 0 (N0) and 75 (N75) min of normoxia, at 15 (H15) and 30 (H30) min of hypoxia, and at 60 (R60) min of recovery. Summary measures analysis was applied to the serial cardiovascular data to focus the number of comparisons [32]. FVR was calculated using Ohm's principle by dividing fetal arterial blood pressure (corrected for amniotic pressure) by femoral blood flow (FBF). Area under the curve was calculated at 30-min intervals (N1, N2, N3, H, R1, R2) for the absolute data describing the fetal cardiovascular responses. Variables were assessed statistically using a two-way ANOVA with RM comparing the effect of time, treatment, and interactions between time and treatment. Where a significant effect of time or treatment was indicated, the *post hoc* Tukey test was used to isolate the statistical differences. For all comparisons, statistical significance was accepted when *P* < 0.05.

## Results

**Fig. 2:**
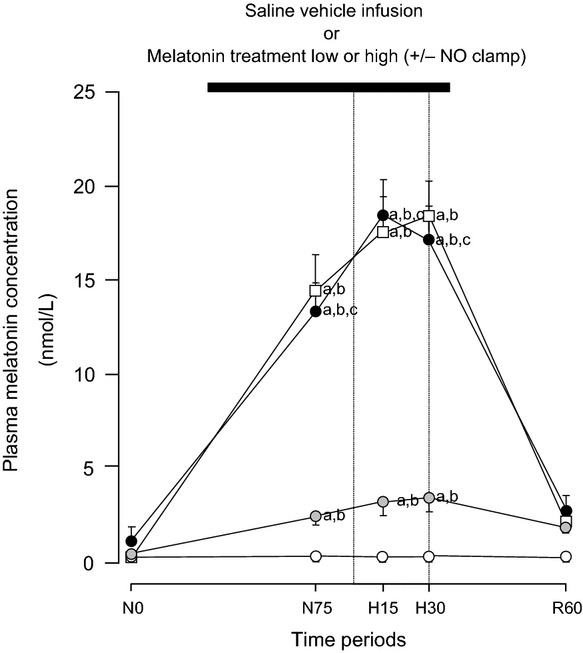
Fetal plasma concentrations of melatonin. Values represent the mean ± S.E.M. for plasma concentrations of melatonin at 0 (N0) and 75 (N75) min of normoxia, at 15 (H15) and 30 (H30) min of hypoxia, and at 30 (R30) and 60 (R60) min of recovery for fetuses exposed to 0.5-hr hypoxia (dashed box) during saline vehicle infusion (○; n = 6), treatment with melatonin low (0.05 ± 0.01 *μ*g/kg/min; 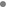; n = 6), treatment with melatonin high (0.5 ± 0.1 *μ*g/kg/min; •; n = 6), or treatment with melatonin high during nitric oxide (NO) blockade with the NO clamp (□; n = 6). Significant differences: ^a^*P* < 0.05, versus time period N0; ^b^*P* < 0.05, versus saline vehicle infusion; ^c^*P* < 0.05, melatonin low versus melatonin high (two-way RM ANOVA with *post hoc* Tukey test).

Basal values for plasma concentrations of melatonin in the fetal circulation were below the detection limit of the assay (0.1 nmol/L). During normoxia, treatment with melatonin elevated fetal plasma concentrations of the indole amine in a dose-dependent manner, with low doses of melatonin treatment achieving concentrations of 2.5 ± 0.4 nmol/L and high doses of melatonin treatment achieving concentrations of 13.3 ± 1.6 nmol/L (Fig. 2). Neither acute hypoxia nor the application of the NO clamp affected significantly the magnitude of the rise in fetal plasma concentrations of melatonin during basal conditions (Fig. 2). At the end of all treatments, fetal plasma concentrations of melatonin returned toward pre-infusion levels (Fig. 2).

Basal values for fetal arterial blood gas status were similar in all fetuses and were within the normal range for the Welsh Mountain sheep fetus at *ca*. 130 days of gestation (Table 1). Infusion with saline vehicle, treatment with melatonin low, treatment with melatonin high, or treatment with melatonin high during NO blockade had no effect on basal arterial blood gas status (Table 1). In all fetuses, acute hypoxia induced significant falls in P_a_O_2_, in Sat Hb, and in pH_a_, without any alteration to P_a_CO_2_ (Table 1). The magnitude of the changes in P_a_O_2_ and Sat Hb were similar in all fetuses. In contrast, the decrement from baseline in fetal pH_a_ during acute hypoxia with saline vehicle infusion (−0.07 ± 0.01) was enhanced during treatment with melatonin high during NO blockade (−0.09 ± 0.01; *P* < 0.05; Table 1). In addition, the maximal increment in fetal [Hb] during acute hypoxia with saline vehicle infusion (2.3 ± 0.3 g/dL) was significantly diminished during treatment with melatonin low (1.0 ± 0.1 g/dL), treatment with melatonin high (0.7 ± 0.2 g/dL), and treatment with melatonin high during NO blockade (1.2 ± 0.2 g/dL; Table 1). During recovery, pH_a_ remained significantly depressed in all fetuses, whereas P_a_O_2_, Sat Hb, and [Hb] returned toward basal values (Table 1).

**Table 1:** Fetal arterial blood gas and acid base status

	Saline vehicle infusion or treatment with melatonin low or high (+/− NO clamp)						
	Normoxia		Acute hypoxia			Recovery	
	N0	N75	H5	H15	H30	R30	R60
pH_a_							
Saline infusion	7.35 ± 0.01	7.3 ± 0.01	7.3 ± 0.01	7.31 ± 0.01^a^	7.28 ± 0.01^a^	7.29 ± 0.01^a^	7.32 ± 0.01^a^
Melatonin (low) infusion	7.3 ± 0.01	7.3 ± 0.01	7.34 ± 0.01	7.32 ± 0.01^a^	7.29 ± 0.01^a^	7.30 ± 0.01^a^	7.33 ± 0.01^a^
Melatonin (high) infusion	7.35 ± 0.01	7.35 ± 0.01	7.34 ± 0.01	7.31 ± 0.01^a^	7.30 ± 0.01^a^	7.30 ± 0.01^a^	7.32 ± 0.01^a^
Melatonin (high) infusion and NO clamp	7.34 ± 0.01	7.34 ± 0.01	7.33 ± 0.01^b^	7.29 ± 0.01^a^,^b^	7.25 ± 0.01^a^,^b^	7.2 ± 0.01^a^,^b^	7.28 ± 0.01^a^,^b^
P_a_CO_2_ (mmHg)							
Saline infusion	54.8 ± 1.0	54.8 ± 1.5	53.7 ± 0.8	53.2 ± 0.5	53.7 ± 1.1	54.0 ± 1.0	54.2 ± 1.0
Melatonin (low) infusion	55.7 ± 1.2	54.8 ± 1.4	54.2 ± 0.5	54.7 ± 0.6	54.0 ± 0.4	54.3 ± 0.8	54.8 ± 1.4
Melatonin (high) infusion	56.0 ± 1.0	56.5 ± 0.9	55.2 ± 0.9	56.2 ± 1.0	53.4 ± 1.3	54.4 ± 1.3	55.2 ± 1.2
Melatonin (high) infusion and NO clamp	56.2 ± 0.9	55.3 ± 1.0	54.0 ± 0.9	54.8 ± 0.7	54.7 ± 0.7	54.3 ± 1.0	54.8 ± 1.0
P_a_O_2_ (mmHg)							
Saline infusion	20.8 ± 0.4	20.5 ± 0.3	10.3 ± 0.6^a^	10.7 ± 0.6^a^	11.5 ± 0.2^a^	21.0 ± 0.6	20.7 ± 0.2
Melatonin (low) infusion	20.5 ± 0.6	20.8 ± 0.5	10.8 ± 0.4^a^	11.0 ± 0.3^a^	11.7 ± 0.2^a^	20.3 ± 0.6	20.3 ± 0.8
Melatonin (high) infusion	21.2 ± 0.5	20.5 ± 0.5	10.0 ± 0.4^a^	11.2 ± 0.3^a^	11.5 ± 0.4^a^	20.0 ± 0.5	20.2 ± 0.5
Melatonin (high) infusion and NO clamp	21.2 ± 0.9	21.3 ± 0.8	10.8 ± 0.4^a^	11.8 ± 0.3^a^	12.2 ± 0.4^a^	21.0 ± 0.9	21.3 ± 0.8
Sat Hb (%)							
Saline infusion	56.0 ± 1.4	55.5 ± 1.7	27.8 ± 1.8^a^	25.7 ± 1.5^a^	27.2 ± 0.9^a^	54.8 ± 2.1	53.9 ± 0.9
Melatonin (low) infusion	54. ± 2.2	55.2 ± 2.1	27.2 ± 1.3^a^	27.1 ± 0.9^a^	27. ± 1.2^a^	52.7 ± 2.5	51.0 ± 2.7
Melatonin (High) Infusion	53.2 ± 1.8	52.1 ± 1.9	25.7 ± 0.7^a^	25.4 ± 0.8^a^	26.3 ± 1.4^a^	51.7 ± 1.2	52.7 ± 1.0
Melatonin (high) infusion and NO clamp	56.7 ± 3.1	56.4 ± 3.3	25. ± 1.7^a^	28.2 ± 2.2^a^	29.4 ± 2.1^a^	52.7 ± 3.8	55.5 ± 2.0
[Hb] (g/dL)							
Saline infusion	9.2 ± 0.1	9.3 ± 0.1	11.2 ± 0.3^a^	11.8 ± 0.3^a^	11.4 ± 0.5^a^	9.7 ± 0.3	9.8 ± 0.3
Melatonin (low) infusion	8.9 ± 0.4	8.8 ± 0.3	9.9 ± 0.4^a^,^b^	9.9 ± 0.4^a^,^b^	9.8 ± 0.5^a^,^b^	8.8 ± 0.6	8.9 ± 0.6
Melatonin (high) infusion	9.2 ± 0.5	9.1 ± 0.6	9.7 ± 0.6^a^,^b^	9.9 ± 0.7^a^,^b^	9.7 ± 0.7^a^,^b^	9.0 ± 0.7	8.9 ± 0.6
Melatonin (high) infusion and NO clamp	8.7 ± 0.4	8.7 ± 0.4	9.4 ± 0.4^a^,^b^	9. ± 0.4^a^,^b^	9.9 ± 0.5^a^,^b^	8. ± 0.3	8. ± 0.3

pH_a_, arterial pH; P_a_CO_2_, arterial partial pressure of CO_2_; P_a_O_2_, arterial partial pressure of O_2_; Sat Hb, percentage saturation of hemoglobin with oxygen; [Hb], blood hemoglobin concentration.

Values represent the mean ± S.E.M. at 0 (N0) and 75 (N75) min of normoxia, at 5 (H5), 15 (H15), and 30 (H30) min of hypoxia, and at 30 (R30) and 60 (R60) min of recovery for fetuses exposed to 0.5 hr of hypoxia during saline vehicle infusion (n = 6), treatment with melatonin low (0.05 ± 0.01 *μ*g/kg/min; n = 6), treatment with melatonin high (0.5 ± 0.1 *μ*g/kg/min; n = 6), or treatment with melatonin high during nitric oxide (NO) blockade with the NO clamp (n = 6).

a Significant differences: ^a^*P* < 0.05, versus time period N0.

b *P* < 0.05, versus saline vehicle infusion (two-way RM ANOVA with *post hoc* Tukey test).

**Fig. 3:**
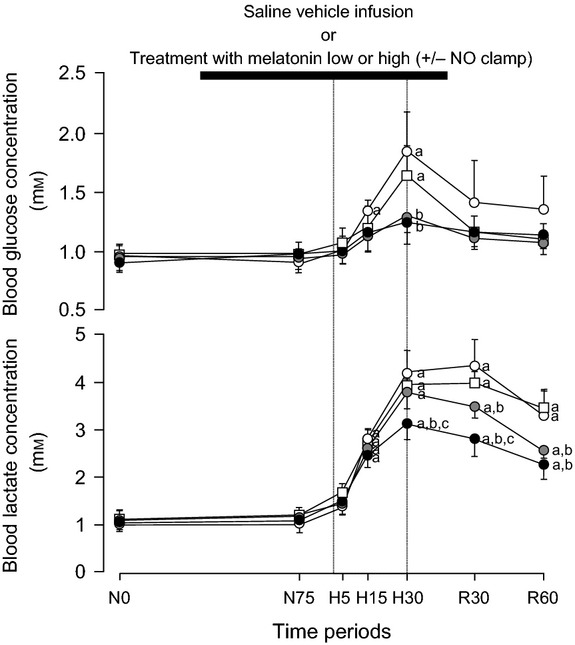
Fetal metabolic responses to acute hypoxia. Values represent the mean ± S.E.M. for concentrations of blood glucose and lactate at 0 (N0) and 75 (N75) min of normoxia, at 5 (H5), 15 (H15), and 30 (H30) min of hypoxia, and at 30 (R30) and 60 (R60) min of recovery for fetuses exposed to 0.5-hr hypoxia (dashed box) during saline vehicle infusion (○; n = 6), treatment with melatonin low (0.05 ± 0.01 *μ*g/kg/min; 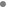; n = 6), treatment with melatonin high (0.5 ± 0.1 *μ*g/kg/min; •; n = 6), or treatment with melatonin high during nitric oxide (NO) blockade with the NO clamp (□; n = 6). Significant differences: ^a^*P* < 0.05, versus time period N0; ^b^*P* < 0.05, versus saline vehicle infusion; ^c^*P* < 0.05, melatonin low versus melatonin high (two-way RM ANOVA with *post hoc* Tukey test).

Basal values for fetal concentrations of blood glucose and lactate were similar in all fetuses and were within the normal range for the Welsh Mountain sheep fetus at *ca*. 130 days of gestation (Fig. 3). Infusion with saline vehicle, treatment with melatonin low, treatment with melatonin high, or treatment with melatonin high during NO blockade had no effect on basal metabolic status (Fig. 3). Acute hypoxia during saline vehicle infusion induced significant increments from baseline in blood glucose (0.85 ± 0.35 mmol/L) and lactate (3.15 ± 0.40 mmol/L) concentrations. In contrast, treatment with melatonin low (0.33 ± 0.10 mmol/L) and treatment with melatonin high (0.29 ± 0.10 mmol/L) significantly diminished the glycemic response to acute hypoxia compared to saline vehicle-infused controls. In addition, treatment with melatonin high (2.07 ± 0.21 mmol/L), but not treatment with melatonin low (2.72 ± 0.23 mmol/L), significantly attenuated the lactic acidemic response to hypoxia. Treatment with melatonin high during NO blockade recovered both the glycemic and lactic acidemic responses toward control values as seen with saline vehicle infusion (Fig. 3). During recovery, blood glucose concentrations returned toward basal values, while blood lactate concentrations remained significantly elevated in all fetuses (Fig. 3).

**Fig. 4:**
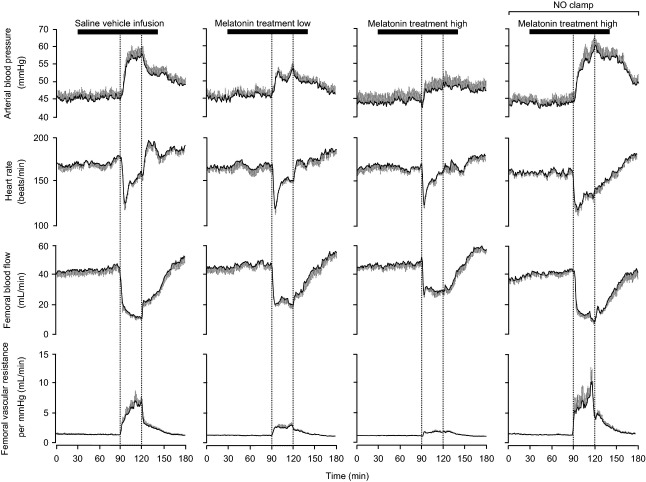
Fetal cardiovascular responses to acute hypoxia. Values represent the mean ± S.E.M. calculated every minute for arterial blood pressure, heart rate, femoral blood flow, and femoral vascular resistance during 1.5-hr of normoxia, 0.5-hr of hypoxia (dashed box), and 1-hr of recovery for fetuses during saline vehicle infusion (n = 6), treatment with melatonin low (0.05 ± 0.01 *μ*g/kg/min; n = 6), treatment with melatonin high (0.5 ± 0.1 *μ*g/kg/min; n = 6), or treatment with melatonin high during nitric oxide (NO) blockade with the NO clamp (n = 6).

**Fig. 5:**
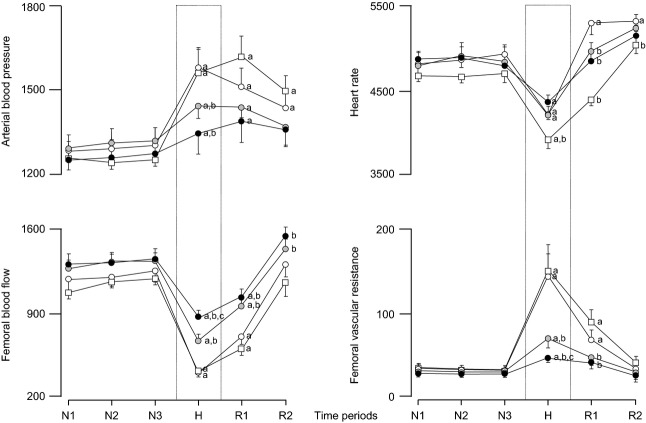
Statistical summary of the fetal cardiovascular responses to acute hypoxia. Values for the statistical summary of the cardiovascular responses represent the mean ± S.E.M. for the area under the curve over every 30 min during normoxia (N), hypoxia (H), and recovery (R) for fetuses during saline vehicle infusion (○; n = 6), treatment with melatonin low (0.05 ± 0.01 *μ*g/kg/min; 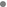; n = 6), treatment with melatonin high (0.5 ± 0.1 *μ*g/kg/min; •; n = 6), or treatment with melatonin high during nitric oxide (NO) blockade with the NO clamp (□; n = 6). Significant differences: ^a^*P* < 0.05, versus time period N1; ^b^*P* < 0.05, versus saline vehicle infusion; ^c^*P* < 0.05, melatonin low versus melatonin high (two-way RM ANOVA with *post hoc* Tukey test).

Basal values for fetal arterial blood pressure, heart rate, FBF, and FVR were similar in all fetuses (Figs 4 and 5). Infusion with saline vehicle, treatment with melatonin low, treatment with melatonin high, and treatment with melatonin high during NO blockade had no effect on basal cardiovascular function (Figs 4 and 5). Acute hypoxia during saline vehicle infusion induced significant increments from baseline in fetal arterial blood pressure (10.0 ± 1.8 mmHg) and FVR (5.27 ± 0.99 mmHg (mL/min)) and significant falls in fetal heart rate (−20 ± 6 beats/min) and FBF (−27 ± 3 mL/min; Figs 4 and 5). In contrast, treatment with melatonin low (5.4 ± 0.7 mmHg) and treatment with melatonin high (4.0 ± 0.8 mmHg) significantly attenuated the magnitude of the mean pressor response to acute hypoxia compared to saline vehicle-infused controls. In addition, treatment with melatonin low (FBF: −21 ± 3 mL/min; FVR: 2.51 ± 0.40 mmHg/(mL/min)) and treatment with melatonin high (FBF: −16 ± 2 mL/min; FVR: 0.99 ± 0.18 mmHg/(mL/min)) significantly attenuated the magnitude of the mean femoral vasoconstrictor response to acute hypoxia in a dose-dependent manner (Figs 4 and 5). Treatment with melatonin high during NO blockade recovered the magnitude of both the mean pressor response (10.5 ± 1.6 mmHg) and the mean femoral vasoconstrictor response (FBF: −27 ± 3; FVR: 5.87 ± 1.35 mmHg/(mL/min)) toward control values as seen with saline vehicle infusion (Figs 3 and 4). The bradycardic response to acute hypoxia was not affected by treatment with melatonin low or treatment with melatonin high; however, the area under the fetal heart rate curve was significantly enhanced during treatment with melatonin high during NO blockade compared to saline vehicle-infused controls (Figs 4 and 5). Treatment with melatonin low, treatment with melatonin high, and treatment with melatonin high during NO blockade also prevented the rebound tachycardia, which was observed in saline vehicle-infused fetuses after the hypoxic challenge (Figs 4 and 5). During recovery, femoral blood flow and vascular resistance returned toward basal values by the end of the experimental protocol in all fetuses. In contrast, while fetal arterial blood pressure and heart returned toward basal values in melatonin low- and melatonin high-treated fetuses, these variables remained significantly altered from basal values in melatonin high during NO blockade (Figs 4 and 5).

**Fig. 6:**
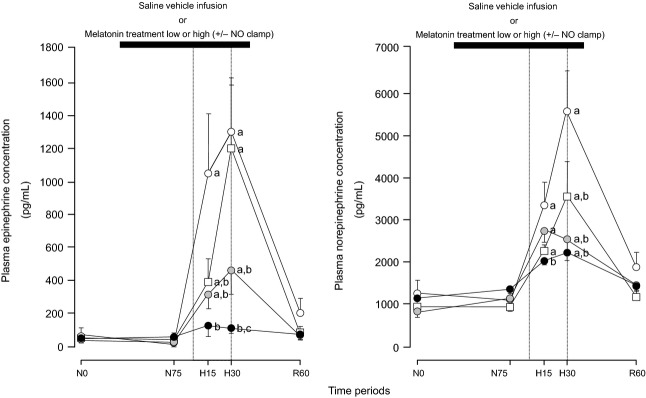
Fetal plasma catecholamine concentrations in response to acute hypoxia. Values represent the mean ± S.E.M. for plasma concentrations of epinephrine and norepinephine at 0 (N0) and 75 (N75) min of normoxia, at 15 (H15) and 30 (H30) min of hypoxia, and at 60 (R60) min of recovery for fetuses exposed to 0.5-hr hypoxia (dashed box) during saline vehicle infusion (○; n = 6), treatment with melatonin low (0.05 ± 0.01 *μ*g/kg/min; 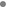; n = 6), treatment with melatonin high (0.5 ± 0.1 *μ*g/kg/min; •; n = 6), or treatment with melatonin high during nitric oxide (NO) blockade with the NO clamp (□; n = 6). Significant differences: ^a^*P* < 0.05, versus time period N0; ^b^*P* < 0.05, versus saline vehicle infusion; ^c^*P* < 0.05, melatonin low versus melatonin high (two-way RM ANOVA with *post hoc* Tukey test).

Basal values for fetal plasma concentrations of epinephrine and norepinephrine were similar in all fetuses (Fig. 6). Infusion with saline vehicle, treatment with melatonin low, treatment with melatonin high, and treatment with melatonin high during NO blockade had no effect on basal catecholamine concentrations (Fig. 6). Acute hypoxia during saline vehicle infusion induced significant increments in both concentrations of epinephrine (1243 ± 392 pg/mL) and norepinephrine (4576 ± 1428 pg/mL; Fig. 6). Treatment with melatonin low (epinephrine: 422 ± 157 and norepinephrine: 1571 ± 286 pg/mL) and treatment with melatonin high (epinephrine: 77 ± 60 and norepinephrine: 1076 ± 124 pg/mL) significantly attenuated the maximal increment in plasma catecholamines compared to saline vehicle-infused controls (Fig. 6). The effect on plasma epinephrine concentrations was dose-dependent (*P* < 0.05). In contrast, treatment with melatonin high during NO recovered the plasma epinephrine response (1156 ± 470 pg/mL) to acute hypoxia back toward control values as seen with saline vehicle infusion. Although a similar effect was observed with the norepinephrine response to acute hypoxia, the recovery was only partial and, hence, the maximal increment (2591 ± 764 pg/mL) was still attenuated compared to saline vehicle-infused controls (*P* < 0.05; Fig. 6). During recovery, plasma concentrations of epinephrine and norepinephrine returned toward basal values in all fetuses (Fig. 6).

## Discussion

The data from the present study show that melatonin treatment diminishes the increase in fetal arterial blood pressure, in FVR, in blood glucose, in blood lactate, and in circulating plasma catecholamines during acute hypoxic stress. These effects of melatonin on stimulated cardiovascular and metabolic functions in the fetus during acute hypoxia were prevented by in vivo NO blockade. Therefore, the data support the hypothesis that melatonin has a significant effect on the fetal defense response to acute hypoxia via increasing NO bioavailability. The use of melatonin in pregnancy may weaken the capacity of the fetus to respond to acute hypoxic stress.

A rapidly accumulating number of studies, including our own, have reported that maternal treatment with melatonin can be protective in early development complicated by conditions of oxidative stress. Richter and colleagues showed that melatonin improves placental efficiency and birthweight and increases the placental expression of antioxidant enzymes in undernourished pregnancy in rats [33]. The laboratory of Vohnnahme [10, 34] has reported that maternal supplementation with melatonin in undernourished pregnancy in sheep improves utero-placental blood flow. The melatonin-induced increase in placental and umbilical arterial blood flow is due to enhanced NO bioavailability [9] and an increase in placental vessel sensitivity to bradykinin-induced relaxation [34]. However, in other vascular beds, such as the coronary circulation, melatonin has also been shown to inhibit NO-induced relaxation by acting on MT(2) receptors and PKG1 to increase PDE5 phosphorylation, thereby decreasing cGMP accumulation in response to NO [35]. Hence, the actions of melatonin appear to change depending on the vascular bed being studied.

Studies by the groups of Tare, Miller, and Herrera [11, 36, 37] have reported that maternal melatonin administration mitigates cerebral oxidative stress, cerebrovascular and endothelial dysfunction, and coronary stiffness in chronically hypoxic and growth-restricted lambs. Tain and colleagues [38] also reported in rats that melatonin therapy prevents programmed hypertension and NO deficiency in offspring exposed to maternal caloric restriction. A collection of other studies has further reported that melatonin and other antioxidants can protect against hypertension, and cardiac and endothelial dysfunction induced by perinatal glucocorticoid therapy [39–42]. Understandably, these long-term protective actions of melatonin have prompted a clinical interest and expedited the design of phase I clinical trials proposing that melatonin might mitigate morbidity and/or mortality in offspring of pregnancy complicated by oxidative stress in humans [13, 43]. However, these longer-term protective actions of melatonin in offspring of pregnancy affected by sustained adverse intrauterine conditions have to be balanced against the possible shorter-term adverse effects of melatonin on the capacity of the fetus to respond to acute hypoxic stress, such as that associated with the imminent labor and delivery of the fetus. This is because a series of studies in our laboratory has reported that an oxidant tone is functional in peripheral resistance circulations of the fetus during late gestation [9, 21–23]. Therefore, treatments with agents that increase NO bioavailability might dampen the fetal peripheral vasoconstrictor response to acute hypoxia, which aids the fetal brain sparing effect.

In this study, we show that treatment with melatonin diminished the fetal in vivo peripheral vasoconstrictor response to acute hypoxia in a dose-dependent manner and that this effect was reverted by in vivo NO blockade. Therefore, the data show that the mechanism underlying the depressive effect of melatonin on fetal peripheral vasoconstriction during acute hypoxia is via increasing NO bioavailability. Interestingly, the doses of melatonin administered to the fetus in the present experiments were between 32 and 320 times lower than those recommended to avoid jet lag in humans. Even at the higher dosing regimen (0.5 ± 0.1 *μ*g/kg/min), the total amount of melatonin given to the fetus during the protocol was *ca*. 0.16 mg compared to the 5.0 mg dose commonly taken to avoid jet lag [30] or the 4.0 mg twice daily dose used in the current clinical trial examining the antioxidant of melatonin in human pregnancies complicated by fetal growth restriction [13].

Additional data in the present study show that treatment of the fetus with melatonin diminished the fetal plasma norepinephrine and epinephrine as well as the fetal metabolic responses to acute hypoxia. Recent studies have shown that melatonin acutely lowers norepinephrine levels in the adult human [44] and gestational melatonin deprivation in sheep results in increased plasma norepinephrine in the newborn [45]; these effects of melatonin may be mediated in part via modulation of central sympathetic outflow [46]. For instance, Wang and colleagues [47] have reported that intracerebroventricular administration of melatonin in pinealectomized animals decreased sympathetic outflow, as measured by a fall in plasma catecholamine concentrations. Central effects of melatonin on sympathetic outflow are supported by melatonin decreasing basal blood pressure only in intact animals, but not in animals with spinal transection [48]. Other studies have reported that melatonin treatment leads to a reduced activity of phenylethanolamine-N-methyl transferase and monoamine oxidase, adrenal medullary enzymes required for catecholamine synthesis [49].

The fetal lactic academic response to acute hypoxia in control fetuses arises from the anaerobic metabolism of glucose in hypoxic fetal tissues, in particular the hind limbs in which blood flow is markedly reduced. Therefore, the attenuated fetal plasma lactic acidemic response to hypoxia in fetuses treated with melatonin is likely due to the diminished fetal peripheral vasoconstrictor and hyperglycemic responses to acute hypoxia [50]. The fetal hyperglycemia increases glucose availability to fetal tissues during acute hypoxia and normally results from a decrease in glucose uptake and utilization by peripheral tissues, coupled with an increase in hepatic glucose production [51, 52]. As fetal treatment with the *α*-adrenergic antagonist, phentolamine, prevented the glycemic response but enhanced insulin secretion during hypoxia [51], both the reduction in insulin-dependent glucose uptake and the increase in glucose production by the fetal tissues may be mediated via sympathetic *α*-adrenergic pathways under these circumstances. Therefore, in the present study, suppression of the fetal glycemic response to acute hypoxia during fetal treatment with melatonin may represent an effect on insulin release and/or on the glucogenic pathways mediated either by sympathetic fibers and/or via elevations in circulating catecholamines. Either way, a negative contribution of melatonin to the activation of the sympathetic adrenomedullary systems during acute hypoxic stress in the late gestation sheep fetus is supported.

Following the end of melatonin treatment, a rapid fall in plasma melatonin concentrations was noted in all fetuses. This is not surprising considering that the half-life of melatonin in fetal ovine plasma is *ca*. 25 min [53]. As melatonin can also cross the placenta [54–56], it may also enter the maternal compartments where its half-life has been reported to be as short as 2 min [53]; however, maternal blood samples were not taken in the present set of experiments. The primary site of melatonin metabolism is the liver, where it is first hydroxylated and then it is excreted via the kidney as 6-hydroxymelatonin sulfate, and to a lesser extent, as 6-hydroxymelatonin glucuronide [57]. Due to the short half-life of melatonin, it is unlikely that fetal treatment with melatonin on one experimental day would affect basal cardiovascular function on the next experimental day. However, to ensure the lack of possible effects of pre-exposure to melatonin on basal cardiovascular function, all experiments were conducted in a randomized order in each of the animals, on consecutive days.

One final consideration is how manipulation of the fetal vascular oxidant tone by agents that increase NO bioavailability, such as melatonin, may affect vascular beds other than the peripheral resistance circulations during acute hypoxic stress. There is robust evidence that the cerebrovascular response to acute hypoxia involves active vasodilatation induced in part via NO-dependent mechanisms [58]. In this context, pregnancies treated with melatonin increasing NO bioavailability may on the one hand oppose circulations that normally constrict during acute hypoxia, such as the femoral vascular bed. On the other hand, melatonin may actually maintain or enhance blood flow in fetal circulations that normally dilate during acute hypoxia, such as the cerebral vascular bed. Under conditions of increased NO bioavailability induced by fetal exposure to antioxidants, the fetal cardiovascular strategy to withstand acute hypoxia may therefore switch to increase cardiac output and maintain perfusion pressure, in many ways, akin to the adult cardiovascular response to acute hypoxia [59]. Studies that include parallel measurement of peripheral and cerebral blood flow in the sheep fetus during acute hypoxia in the presence of antioxidant treatment in doses that are administered to humans in healthy pregnancy as well as pregnancy complicated by adverse intrauterine conditions are therefore essential to establish the balance of potential beneficial and adverse effects of fetal exposure to melatonin.

In summary, melatonin has direct effects on the fetal cardiovascular and metabolic defenses to acute hypoxia in a dose-dependent manner via mechanisms involving increased NO bioavailability and diminished plasma catecholamine responses.
